# An algorithmic approach to gastrointestinal bleeding in patients receiving antithrombotic agents 

**Published:** 2020

**Authors:** Amir Sadeghi, Mohammad Reza Zali, Hamid Mohaghegh Shalmani, Pardis Ketabi Moghadam, Mohsen Rajabnia Chenari, Mohammad Ali Karimi, Sina Salari, Hamid Asadzadeh-Aghdaei

**Affiliations:** 1 *Gastroenterology and Liver Diseases Research Center, Research Institute for Gastroenterology and Liver Diseases, Shahid Beheshti University of Medical Sciences, Tehran, Iran*; 2 *Basic and Molecular Epidemiology of Gastrointestinal Disorders Research Center, Research Institute for Gastroenterology and Liver Diseases, Shahid Beheshti University of Medical Sciences, Tehran, Iran*; 3 *Taleghani Hospital, Shahid Beheshti University of Medical Sciences, Tehran, Iran *

**Keywords:** Algorithms, Gastrointestinal bleeding, Thromboembolic events, Antithrombotic agents

## Abstract

Gastrointestinal bleeding is an overwhelming complication of patients taking antithrombotic agents. These drugs pose a challenge to physicians in the management of bleeding to establish hemostasis without putting these patients at a higher risk for thromboembolism. This study aims to propose an algorithmic approach to four major groups of patients receiving antithrombotic agents (single antiplatelet agents, dual antiplatelet agents, anticoagulants and direct oral anticoagulants) to decide when and how these drugs should be held or restarted to offset between the risk of re-bleeding and thromboembolism. Four case-based algorithms are proposed in this article based on some relevant articles. Having designed four case-based algorithms, we are hoping to guide physicians who face a dilemma on the management of patients receiving antithrombotics when gastrointestinal bleeding occurs. Patients using antithrombotics referred for gastrointestinal bleeding were stratified into four groups based on the medication which is used as an antithrombotic agent and four algorithms were designed which are presented here. We have made an attempt to have a stepwise approach to four cases relevant to the study and have an evaluation on the management of their antithrombotic agents during an episode of gastrointestinal bleeding. It is widely accepted that antithrombotic agents should be restarted as soon as possible after the establishment of hemostasis in a patient taking antithrombotics referring for gastrointestinal bleeding. The time for resuming these drugs is different based on the severity of bleeding, the probability of thromboembolic events, and the nature of the antithrombotic medication which is used by the patient.

## Introduction

 Management of gastrointestinal (GI) bleeding for patients taking antithrombotic agents including antiplatelet drugs, anticoagulants and direct oral anticoagulants (DOACs) has always been challenging. Although there is a general agreement on the management of active bleeding in this situation, more studies are required to come to a consensus as to when and how antithrombotic agents should be reintroduced. To make restarting these antithrombotic agents justifiable in a patient with recent gastrointestinal bleeding, the risk of re-bleeding in contrast to the benefit of a decrease in the percentage of thrombotic events should be assessed individually. Base on the revised guidelines, the benefit of reinstating these agents outweigh the risk of withholding them for a long time (1,2). The risk of thromboembolism and also the risk of bleeding in patients on antithrombotic agents who referred for GI bleeding in this guideline review is stratified based on previous studies (2-4). The characteristics of a major GI bleeding is shown in table-1. It should be noted that one of these criteria is sufficient to label a bleeding event as a major bleeding (2,5). High-risk thromboembolic events are presented in table-2. One of the situations mentioned in table-2 is required to categorize a patient in the high-risk thromboembolic events (2,5,6). In the present study, we have attempted a case-based approach to the management of patients receiving antithrombotic agents who referred for gastrointestinal bleeding based on the risk stratification for GI bleeding and thromboembolism as depicted in table-1&2.

**Table 1:** Characteristics of a major GI bleeding

Vomiting or passage of a large amount of fresh blood, maroon blood or black tarry stool through the rectum,
Shock and hemodynamic instability,
A sudden drop in hemoglobin level to 6 gr/dl or less,
Requiring transfusion of at least 4 units of packed RBCs,
Bleeding continuing for at least 3 days,
A significant re-bleeding in 1 week,
High risk stigmata of bleeding in endoscopy including lesions with active bleeding (spurting or oozing), non-bleeding visible vessels, adherent clots.

**Table 2:** Patients of high-risk thromboembolic events

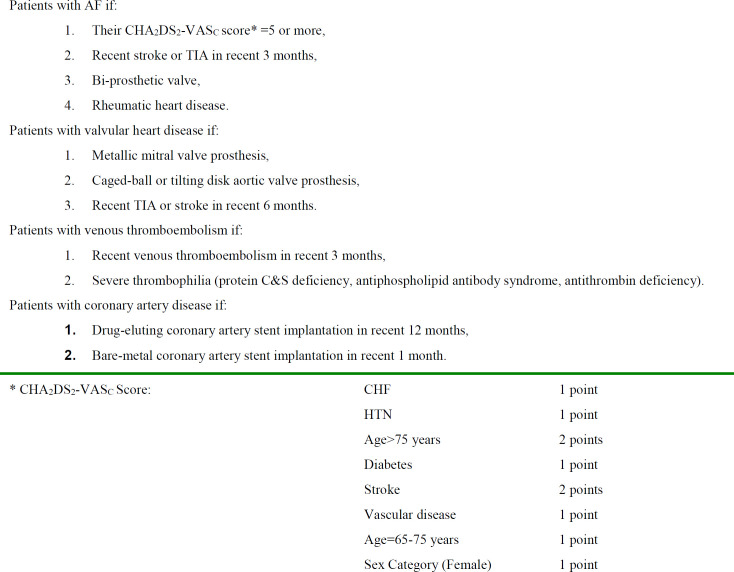

**Algorithms**

***Patients on dual antiplatelet therapy***

1. A 67-year-old man presented to the general gastroenterology clinic with complaints of profuse melena for 3 days. He had a history of coronary artery disease with earlier percutaneous coronary intervention with drug-eluting stent implantation 5 months before. Then, he was started on a dual antiplatelet therapy with aspirin and clopidogrel. On arrival, he was hemodynamically unstable with hypotension, tachycardia and clouding of consciousness. Before transfusion, Hb level was 5.9 gr/dl. Besides standard medical treatments, an examination with flexible endoscope was performed which was notable for a large ulcer with oozing in antrum. Esophagus, stomach and duodenum were all full of blood and blood clot. Initially, diluted epinephrine (1/10,000) was injected and then 2 endoscopic hemoclips were inserted and oozing stopped.

**Figure 1:**
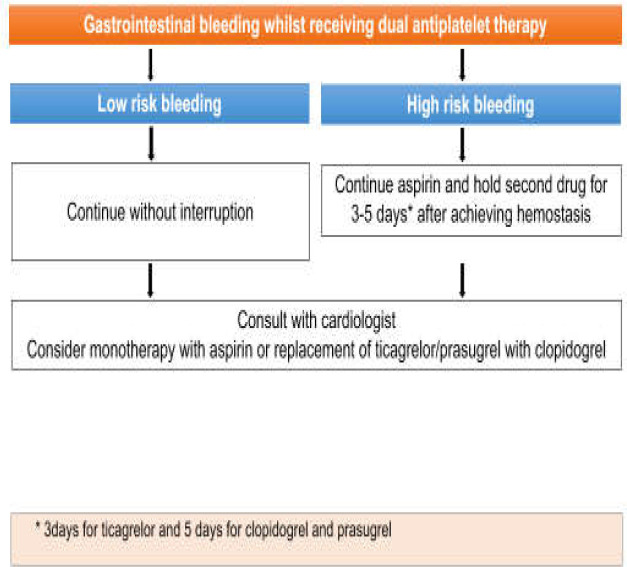
The algorithm for the management of GI bleeding in patients taking dual antiplatelet therapy

Antiplatelet agents are used as primary and secondary preventive drugs of choice for arterial thrombosis in patients with certain medical conditions to prevent disastrous consequences in coronary artery, as well as cerebrovascular and peripheral vascular diseases. Dual antiplatelet therapy using aspirin (a non-selective cyclooxygenase inhibitor) plus a P2Y12 inhibitor such as clopidogrel, ticagrelor or prasugrel is suggested for especial situations in order to obtain a better antiplatelet effect than it is anticipated by single antiplatelet therapy (7). Patients with coronary artery disease with a history of stent placement are at risk for thrombosis and restenosis particularly during the first months of a drug-eluting stent placement and the first month of a bare-metal stent implantation. Generally, it is accepted that a combined antiplatelet therapy should be continued for 12 months in patients with a drug-eluting, and 1 month for those with a bare-metal stent.(8) GI bleeding is an overwhelming complication of dual antiplatelet therapy leading to withholding antiplatelet drugs. Generally, withholding both antiplatelet agents simultaneously is not recommended. The median time to coronary stent thrombosis is estimated as short as 7 days for the discontinuation of both drugs compared with 122 days for only clopidogrel withholding (9). Overall, it is recommended that aspirin be continued and Y2P12 inhibitors be omitted during an acute phase of a major GI bleeding because of an interaction between this class of medication with proton pump inhibitors (PPIs). Both PPIs and clopidogrel are metabolized by CY2C19 pathway (10). The point is when and how we can reintroduce these agents to offset the risk and benefit of restarting antiplatelet agents. The second drug is suggested to be reintroduced 3-5 days after discontinuation. Ticagrelor is preferably started 3 days after hemostasis but restarting of clopidogrel and prasugrel would be deferred up to 5 days. This difference is attributed to the mechanism of action of ticagrelor in contrast to clopidogrel and prasugrel which is a reversible P2Y12 receptor inhibitor with a platelet function returning of about 3–5 days after discontinuation. The latter drugs are irreversible inhibitors of P2Y13 receptor requiring at least 5-7 days for platelet function recovery (11-13) Studies have shown that initiation of antiplatelet agents in this stage would decrease the risk of restenosis although it does not increase the risk of re-bleeding (1,2). There is a general agreement on the continuation of these drugs if GI bleeding is minor (11). Vorapaxar, a new anti-platelet agent, is a protease-activated receptor (PAR-1) antagonist that inhibits thrombin receptor. It is indicated in patients with a history of myocardial infarction or peripheral arterial disease but is contraindicated in patients with a history of stroke, transient ischemic attack or intracranial hemorrhage because of the risk of intracranial hemorrhage. Besides these suggestions for acute phase of bleeding, it is suggested that antiplatelet agents be withdrawn for patients using them as primary prevention under supervision of a cardiologist as a long-term therapy. Trials in Japan on primary prevention of cardiovascular events by aspirin have not shown any benefit for aspirin in the reduction of cardiovascular events (14). It is in contrast to the study of Souk et al. which shows a protective effect even beyond cardiovascular benefits for aspirin usage as a primary prophylaxis for coronary artery diseases and cerebrovascular diseases in non-variceal GI bleedings (15). The present study is in line with the first approach and recommends discontinuation of aspirin as a primary prophylaxis if possible. It is also recommended that aspirin be prescribed with a lower dose. Furthermore, it is suggested that patients on ticagrelor or prasugrel as more potent antithrombotic agents but with a higher risk of bleeding be switched to the clopidogrel (16,6). PPI consumption and H.pylori eradication should be taken into consideration too. Initial usage of IV PPI after upper gastrointestinal bleeding is highly recommended by ESGE guideline and it is advised that PPI infusion be continued for 72 hours from gastrointestinal bleeding due to anti-platelet therapy. Oral PPI therapy should be considered after reinitiating antiplatelet therapy (17). Management of GI bleeding in patients receiving dual antiplatelet therapy has been depicted in figure-1. Based on the mentioned tips, the presented case whose clinical scenario and endoscopic findings were suggestive of a major bleeding (unstable hemodynamic and peptic ulcer disease with oozing) in the background of a high probability for thromboembolic events (implantation of drug-eluting stent in the last 5 months) drove us to withhold clopidogrel and continue aspirin. After resuscitation with IV fluids, endoscopic hemostasis was successfully performed. He was transferred to ICU and went on IV PPI therapy. Three days after hemostasis, he was not found to have an Hb drop and he continued to remain stable. A second-look endoscopy revealed inserted hemoclips without bleeding. He was sent to general gastroenterology ward and was restarted on clopidogrel after 5 days of index GI bleeding. H.pylori eradication was considered for him and he was advised to take oral PPI while taking dual antiplatelet therapy.

***Patients on single antiplatelet therapy***

2. A 51-year-old woman was admitted to the emergency department with severe and non-stop hematemesis after a long episode of intractable vomiting unresponsive to antiemetic agents. Her past medical history was remarkable for diabetes mellitus and hypertension. So, she had been advised to take daily low-dose aspirin. On admission, she was noted to be orthostatic and hemodynamically unstable requiring packed cell transfusion. After resuscitation, she was transferred to the endoscopy unit which revealed a mallory weiss tearing with visible vessel in distal part of esophagus and a large amount of fresh blood in the stomach. An endoscopic hemoclip was inserted and no further bleeding was detected. 

**Figure 2:**
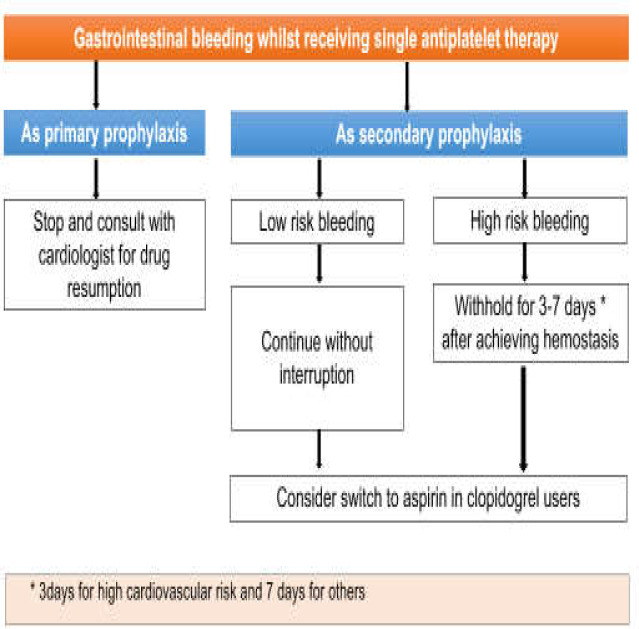
The algorithm for the management of GI bleeding in patients receiving single antiplatelet therapy

The management of a patient receiving single antiplatelet agent is somehow different from patients on dual antiplatelet drugs. A review of relevant studies shows that aspirin can safely be continued in minor GI bleedings. In contrast to minor bleedings, risk-benefit assessment of patients with major GI bleedings has proposed discontinuation of aspirin before endoscopy. Reinstating of aspirin 3-7 days after index bleeding has not added the risk of re-bleeding as well as thrombosis based on recent studies (18). Above that, findings have shown that platelet transfusion has not been improving the outcome of patients receiving single antiplatelet therapy (19). It is reflected in the study of Zakko et al. that platelet transfusion in this group of patients without thrombocytopenia has not reduced the risk of re-bleeding but has increased mortality rate (19). Similar to other patients with GI bleeding, these patients are also encouraged to eradicate H. Pylori infection and take PPI to reduce the risk of re-bleeding in near future (17). The algorithm for the management of GI bleeding in patients receiving single antiplatelet therapy is presented in figure-2. The presented patient had a major and life-threatening GI bleeding on aspirin. As evidenced in the previous studies, aspirin as a single antithrombotic agent be withheld during the acute phase of major GI bleeding. She was started on IV fluids and IV PPI infusion (17). An endoscopic hemostasis was considered as soon as possible after resuscitation. Then, our patient was sent to ICU. Having performed the hemostasis, aspirin was restarted in 3 days. A consultation with a cardiologist was demanded to evaluate the possibility of discontinuation of aspirin as a life-long primary preventive drug for cardiovascular and cerebrovascular events. She was discharged from hospital and was advised to take H. pylori eradication regimen and continue oral PPI as long as she was on aspirin (17).

***Patients on warfarin***

3. A 76-year-old woman was referred to the hospital with complaints of hematemesis and melena for the last 2 days. A review of her record presented that she had an episode of transient ischemic attack 2 month before, and hypertension, congestive heart failure, atrial fibrillation and diabetes mellitus for several years. She was taking losartan, carvedilol, spironolactone, furosemide, warfarin and metformin. At the time of arrival, she was conscious and oriented to person, place and time but she was uncomfortable with weak peripheral pulses, irregular tachycardia and hypotension. Her laboratory results were hemoglobin 6.5 mg/dL, creatinine 0.8 mg/dL, INR=3.8. She was resuscitated by IV crystalloids, FFP and packed cell infusion and then she underwent an urgent endoscopy. A duodenal ulcer with a large adherent clot was detected in bulb. A successful hemostasis was achieved by one hemoclip. 

**Figure 3:**
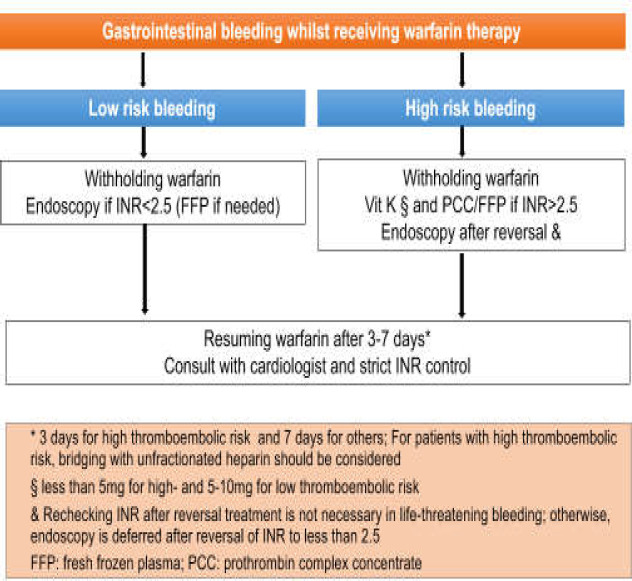
An algorithm for the management of patients receiving warfarin referred for GI bleeding

Warfarin is an accepted oral anticoagulant which inhibits coagulation cascade and prevents thromboembolic events. Besides the benefit of warfarin as an anticoagulant, the risk of bleeding remains a major concern particularly when restarted within 7 days from a bleeding event (20). Although a large number of anticoagulant agents under the name “direct oral anticoagulants (DOAC)” were introduced and approved for clinical usage, warfarin has still its place due to the possibility of strict dose adjustment according to the schedule of INR which is not feasible for DOACs (21). Based on the literature, warfarin should be stopped if bleeding occurred regardless of the severity of bleeding (22). The risk assessment of thromboembolism is navigating whether a bridging therapy with low molecular weight heparin (LMWH) is indicated or not. Patients of high-risk thromboembolic events are highly suggested to receive therapeutic dose of LMWH 48hrs of warfarin hold (23,24). Some authors believe that patients with high-risk stigmata of bleeding should be started on prophylactic dose of LMWH in 48hrs and then on a therapeutic dose in 72hrs (24). Reinitiating warfarin depends on the risk assessment of thromboembolism. Patients in high-risk thromboembolic events are suggested to initiate warfarin on day 3 after hemostasis but it may be postponed for 7 days for patients in low-risk thromboembolism (25). The crucial thing in the management of an acute episode of bleeding among patients receiving warfarin is warfarin reversal by vitamin K and FFP/PCC. In contrast to FFP, PCC does not require ABO matching and has faster onset of action and minimal risk of volume overload. So, usage of 4-factor PCC is superior to FFP in studies (26). Some authors believe that warfarin reversal should be taken into account for each patient with major bleeding regardless of the level of INR. On the other hand, some studies recommend a warfarin reversal in major bleedings with levels of INR more than 2.5 which is higher than the therapeutic level (27). The current guideline emphasizes the latter approach. Warfarin reversal in minor bleedings would only be indicated if the level of INR is higher than 2.5. It is strongly accepted that an urgent endoscopy for life-threatening GI bleedings should not be deferred until the correction of INR. Thus, there is no need to recheck INR after transfusion of required factors for an urgent endoscopy in major bleedings in contrast to endoscopies for minor bleedings which is highly recommended to be delayed up to an INR level of less than 2.5. The reason why vitamin K is usually added to the FFP/PCC for the reversal of warfarin in major bleedings is the short half-life of factor VIIa in these coagulation surrogates which makes oral/IV replacement of vitamin K a necessity to restore its endogenous source (26). Reversal of warfarin for clinically minor bleedings proceeds with FFP alone. Then, the result of endoscopy would provide us with complementary information about the cause of hemorrhage. In this regard, some of the patients with clinically minor bleedings belong to the group of patients with endoscopic high-risk stigmata of bleeding. Approach to this subset of patients should then be directed towards the mentioned approach for major bleedings (27). Findings have shown that a dose of vitamin K as low as 2.5 mg per day is potent for warfarin reversal. However, some other studies suggest a vitamin K dose of less than 5mg for patients categorized as high risk for thromboembolism and 5-10mg for those with low risk characteristics of thromboembolic events (28). We suggest the latter approach that is a low-dose vitamin K of less than 5mg in patients for high-risk thromboembolic events to reduce the risk of thromboembolism and 5-10mg in patients for low-risk thromboembolism. A comprehensive algorithm for the management of patients receiving warfarin referred for GI bleeding is shown in figure 3.

 As reflected in the third case, the patient with AF in addition to risk factors including age>75, history of DM, CHF, HTN and a TIA in recent 3 months has CHA2DS2-Vasc score of 7 which makes her extremely potent for thromboembolism. On the other hand, an unstable hemodynamic in our patient signaled a life-threatening bleeding. This scenario urged for warfarin reversal by FFP and vitamin K, holding warfarin, an urgent endoscopy for hemostasis regardless of the level of INR after reversal, bridging with LMWH in 48 hours, and reinitiating warfarin after at least 3 days from hemostasis. This patient should be advised to have a tighter control of INR level since then, besides H.pylori eradication and long term usage of PPIs (29).

***Patients on direct oral anticoagulants***

4. An 18-year-old man with a history of pulmonary thromboembolism (PTE) 4 months ago. He is currently on rivaroxaban 20 mg/day. His physical examination and his vital signs are all normal. He undergoes an upper endoscopy which demonstrates mild erythema in body and antrum, several superficial clean-based ulcers in antrum and an eroding ulcer with non-bleeding visible vessel in bulb. A successful endoscopic therapy was performed and the patient was transferred to ICU.

**Figure 4:**
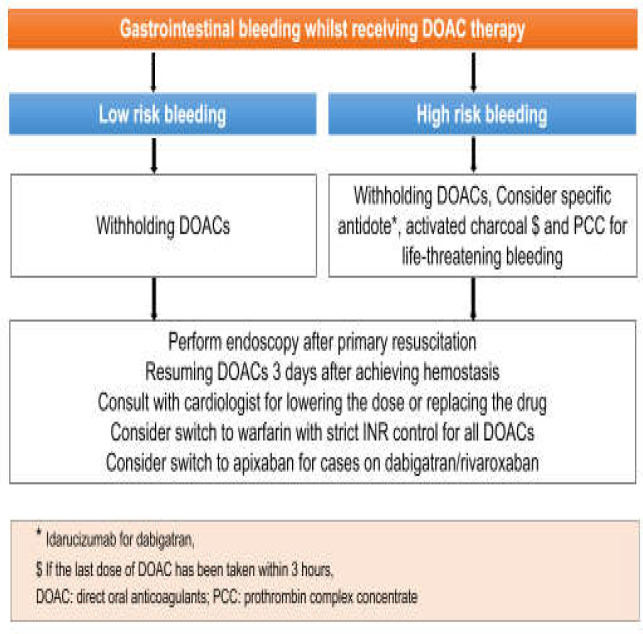
The approach to the GI bleeding in patients receiving DOACs

Usage of DOACs like dabigatran, as a direct thrombin inhibitor and apixaban, and rivaroxaban and edoxaban as direct factor Xa inhibitors, is of great interest among physicians and also patients requiring anticoagulant therapy, due to their dispensable monitoring which obviated the need for dose monitoring (30). Nevertheless, this benefit makes these drugs a greater threat for GI bleeding. Connolly et al. (2009) made a comparison between warfarin and dabigatran indicating an overall increased risk of major GI bleeding for dabigatran in contrast to warfarin (31). The same results have been found for rivaroxaban and apixaban (32). Other studies have indicated that making a comparison between bleeding tendency of warfarin and DOACs regardless of the age and comorbidities could result in a marked bias. As an example, elderly patients on warfarin due to AF have been at higher risk for bleeding in contrast to younger patients on apixaban (25). Thrombin time (TT) is a sensitive test for the effects of dabigatran. It is indicated that there is a correlation between plasma concentration of dabigatran and activated thromboplastin time. Normal TT shows the absence of dabigatran in the blood circulation, so there is no need to eliminate it or its antidote idarucizumab by dialysis (33). There are also some assays tracing anti-factor Xa for rivaroxaban and other drugs of this group which are not accessible to all laboratories and are not suggested. Although coagulation tests like thromboelastography are involved by DOACs, the results are not consistent with drug effect and drug concentration, so they are not used for the effect assessment of DOACs (34,35). In the acute phase of GI bleeding, these drugs should be discontinued. Major bleedings raise a major concern for hemostasis establishment in using DOACs, making drug reversal a necessity in acute phases of a severe GI bleeding. Unfortunately, platelet transfusion, desmopressin, cryoprecipitate, FFP and vitamin K have not been found to be effective in subsiding the effect of these drugs (7). Findings for the effect of PCC have been complicated. PCC has been thriving in the reduction of prolonged PT due to rivaroxaban but unsuccessful in normalizing dabigatran-induced abnormal TT and PTT (36). Idarucizumab (a potent monoclonal antibody directed against dabigatran) is the known antidote of dabigatran (37). Notably, the use of these antidotes should be avoided in minor bleedings due to the risk of thrombosis (38) but during an episode of a major bleeding, PCC and the antidote of dabigatran are suggested. Above that, dabigatran is known to be dialyzable and charcoal is to be considered for the last dose of DOACs ingested in the last 2-4 hours (9,39). Unlike GI bleedings on warfarin, bridging therapy is not recommended for an acute episode of GI bleeding in patients receiving DOACs. Based on the literature following hemostasis, DOACs are to be restarted in 3 days (1). Among them, apixaban has the lowest risk of bleeding so it is recommended that dabigatran and rivaroxaban be switched to apixaban 5 mg twice daily in patients with GI bleeding (39,40). Apixaban could be titrated to the lower dose of twice-daily 2.5 mg in patients with age>80, weight<60 kg and serum creatinine>1.5 mg/dl (39,41,42). DOACs clearance is prolonged in patients with reduced renal clearance especially dabigatran which is mostly (80%) cleared by the kidneys. Edoxaban, rivaroxaban and apixaban have 50%, 33% and 25% renal elimination, respectively. It is recommended that DOACs be used cautiously by patients with severe renal insufficiency (CrCl=15–29 mL/min), and no DOAC should be used by patients with CrCl <15 mL/min (43,44). The hepatic elimination of apixaban, rivaroxaban, edoxaban, and dabigatran is 75%, 65%, 50%, and 20% respectively. These drugs can be used in cirrhotic patients. The fact that they do not need to be monitored by INR makes them favorable for cirrhotic patients who have altered INR and other coagulation tests attributed to the cirrhosis. But, more studies are required to definitely confirm the safety of DOACs in cirrhotic patients (45). The approach to the GI bleeding in patients receiving DOACs is depicted in figure 4. 

The presented case is a young man with a history of recent PTE making him highly susceptible to thromboembolic events. He was admitted with a GI bleeding endoscopically high risk for bleeding. On admission after resuscitation with IV fluids and IV PPI, rivaroxaban was stopped and an endoscopic hemostasis was established urgently. The episode of bleeding had occurred at least 10 hours after the last dose of rivaroxaban was administered, so charcoal was not started. Rivaroxaban was restarted 3 days after hemostasis. Nevertheless, he was advised to substitute rivaroxaban with apixaban (5 mg twice-daily) or warfarin (with a strict dose adjustment based on INR level) under the supervision of his cardiologist. H.pylori eradication regimen and continuation of oral PPI therapy after discharge were ordered (17). 

## Conclusion

Management of GI bleeding which is commonly due to peptic ulcer diseases (duodenal ulcers more than gastric ulcers) (46) in patients receiving antithrombotic agents is of great importance for physicians. Hemostasis establishment in these patients who referred for major GI bleeding is challenging because reversal of anticoagulants and simultaneous discontinuation of antithrombotic agents leave patients prone to thromboembolism. In this study, we have tried to introduce 4 algorithms in the management of patients taking single antiplatelet agents, dual antiplatelet agents, warfarin and direct oral anticoagulants admitted with GI bleeding based on a comprehensive review of relevant articles. Stratification of patients for the severity of GI bleeding and probability of thromboembolism has been performed based on some previous studies (3,4). It has been tried that a step-wise approach be introduced indicating the best time for discontinuation and restarting of antithrombotics in patients with GI bleeding with regard to the risk of thromboembolic events and re-bleeding.

## Conflict of interests

The authors declare that they have no conflict of interest.
